# Does the local food environment around schools affect diet? Longitudinal associations in adolescents attending secondary schools in East London

**DOI:** 10.1186/1471-2458-13-70

**Published:** 2013-01-24

**Authors:** Dianna Smith, Steven Cummins, Charlotte Clark, Stephen Stansfeld

**Affiliations:** 1 Centre for Primary Care and Public Health, Blizard Institute, Queen Mary University of London, Yvonne Carter Building, 58 Turner Street, London, E1 2AB, UK; 2 Department of Social & Environmental Health Research London School of Hygiene & Tropical Medicine, 15-17 Tavistock Place, London, WC1H 9SH, UK; 3 Centre for Psychiatry, Old Anatomy Building, Barts and The London School of Medicine, Queen Mary University of London, Charterhouse Square, London, EC1M 6BQ, UK

**Keywords:** Local food environment, Diet, Geographic information systems (GIS), Adolescents, Schools

## Abstract

**Background:**

The local retail food environment around schools may act as a potential risk factor for adolescent diet. However, international research utilising cross-sectional designs to investigate associations between retail food outlet proximity to schools and diet provides equivocal support for an effect. In this study we employ longitudinal perspectives in order to answer the following two questions. First, how has the local retail food environment around secondary schools changed over time and second, is this change associated with change in diet of students at these schools?

**Methods:**

The locations of retail food outlets and schools in 2001 and 2005 were geo-coded in three London boroughs. Network analysis in a Geographic Information System (GIS) ascertained the number, minimum and median distances to food outlets within 400 m and 800 m of the school location. Outcome measures were ‘healthy’ and ‘unhealthy’ diet scores derived from adolescent self-reported data in the Research with East London Adolescents: Community Health Survey (RELACHS). Adjusted associations between distance from school to food retail outlets, counts of outlets near schools and diet scores were assessed using longitudinal (2001–2005 n=757) approaches.

**Results:**

Between 2001 and 2005 the number of takeaways and grocers/convenience stores within 400 m of schools increased, with many more grocers reported within 800 m of schools in 2005 (p< 0.001). Longitudinal analyses showed a decrease of the mean healthy (−1.12, se 0.12) and unhealthy (−0.48, se 0.16) diet scores. There were significant positive relationships between the distances travelled to grocers and healthy diet scores though effects were very small (0.003, 95%CI 0.001 – 0.006). Significant negative relationships between proximity to takeaways and unhealthy diet scores also resulted in small parameter estimates.

**Conclusions:**

The results provide some evidence that the local food environment around secondary schools may influence adolescent diet, though effects were small. Further research on adolescents’ food purchasing habits with larger samples in varied geographic regions is required to identify robust relationships between proximity and diet, as small numbers, because of confounding, may dilute effect food environment effects. Data on individual foods purchased in all shop formats may clarify the frequent, overly simple classification of grocers as ‘healthy’.

## Background

Extensive research is dedicated to exploring the relationship between residents’ diets and retail food environments around the home. Frequently the aim of these studies is to assess how choice, cost and quality of food (and by extension, diet) or distance to a store may differ in relation to neighbourhood deprivation [1-4], building on decades of interest in the potential relationship between retail food availability and diet [5-8]. Often the analysis involves cross-sectional data about retail food outlets in a neighbourhood, with distance measured from a population centre to each store using road networks in a Geographic Information System (GIS). In addition, product surveys in shops can provide insight on food price, variety and quality to gather more data about how food choices may be constrained by location or economic circumstances [1,6,9].

Recent studies linking diet with local setting incorporate broader definitions of food environments and refined methodologies to better understand the importance of geography to dietary choice. Additional environments where people spend much of their time are considered instead of or alongside residential areas, such as the workplace for adults and schools in the case of children and adolescents [10-16]. Research is emerging which focuses on daily activity space, using global positioning systems (GPS) to capture an accurate and comprehensive assessment of individuals’ food environments [17]. Due to the ethical challenges and time-consuming nature of collecting such detailed travel data, at present most researchers continue to apply GIS methods to assess selected food environments, focussing on the food choices available in different areas [4,11,18].

School food environments are an important alternative environmental exposure for diet outcomes in children and adolescents. Each school day children will eat at least one meal at or near school they attend. A recent School Food Trust report suggested that only 44.1% of primary students and 37.6% of secondary students purchased (or accessed via the Free School Meal [FSM] programme) school lunches in England between 2010–2011 [19]. This statistic suggests that, during the school day, most students are purchasing food for consumption elsewhere. This may be from vending machines within schools, choosing alternative options in school canteens, or sourcing food during the commute to and from school [13,20]. As children age and gain autonomy they are able to exert more independent choice over their food options in these environments compared to those within the home.

A small study in the UK further supports the hypothesis that retail food outlets near to schools are an important source of food for most students. Researchers assessed dietary habits of students using a 5-day food frequency questionnaire (FFQ) at one deprived urban and one affluent suburban secondary school, with results similar to the School Food Trust survey. The authors concluded that of the 332 students surveyed (age 13–15 years, with 16–17 year olds included in the suburban school), 80% purchased food from outlets they classed as ‘fringe’, located near the schools, 68% brought food from home and only 59% purchased food at school [21]. The average number of purchases from fringe shops by urban students was 11.5 times per week, providing further evidence that shops on the way to and from school can be a significant source of food. Observations of student food purchases in the fringe shops (n=587) concluded that these purchases provided 23% of the student’s daily energy needs. These results highlight the importance of local shops in urban deprived areas as a source of food for adolescent students, who report that prices in local shops or takeaways are usually lower than in the schools [21]. A 2005 Sodexho survey of student purchasing patterns found that students spent £1.01 on the way to and from school each day. Students may compare prices and choose to purchase food away from school to save money [22].

When classifying the residential food environment, often a simple stratification of outlet type is applied. Fast food outlets and small corner shops/convenience stores are typically identified as providing ‘unhealthy’ food options such as chocolate, crisps and sugar-sweetened drinks. Grocery stores/supermarkets are usually a proxy for ‘healthy’ food choices, providing a range of fresh produce [10,14,23-25]. Fresh fruit and vegetables are usually less expensive with greater quality and variety available from larger stores while limited shelf space/chiller space and lower turnover can restrict fresh produce stocked in smaller shops [1,2]. However, there are challenges with this classification of food sources. The analysis of products purchased by customers, rather than items available, can help researchers avoid the dichotomy of supermarkets as ‘healthy’ food sources and convenience stores as ‘unhealthy’; this categorisation ignores the wide range of unhealthy foods available at most if not all supermarkets. Diet surveys such as FFQs can collect data on individuals’ diets, particularly fruit and vegetable consumption, fizzy drinks and fast food. This provides a more direct measure of food consumed, in contrast to in-store surveys of food available for purchase [16,26,27]. Much of the previous research on residential food environments has implicitly focussed on food purchased for later preparation at home, while non-residential retail food environments may be more influential in the purchase of ready to eat foods for groups such as students on the way to/from school.

Most studies investigating food environments have been cross-sectional out of necessity; longitudinal studies assessing a causal link between food environment and diet are rare [25,28]. Results published from longitudinal studies, mainly based in the US, are mixed. One study showed that lower income men may consume more fast food when these outlets are located nearer to their homes, there was no clear indication that proximity to grocery stores affected fruit and vegetable consumption [25]. A study of young adults indicated no association between residential fast food access and consumption, though the authors suggest that food options near work or school should be evaluated in addition to areas around the home [28]. Similar to research on diet and retail environments, few longitudinal studies have studied the potential relationship between health and retail food availability [29-31]. A long-term study of the relationship between food environment and body mass index (BMI) over 30 years followed 3,113 people from 1971–2001. As with other studies, results did not clearly show a significant relationship between proximity (measured by driving distance) to a range of food outlet types and individual BMI [29].

The benefit of longitudinal analysis is the ability to follow the same individuals over time, tracking the changing food environment and their health to identify any effect food environment may have after controlling for individual characteristics. While cross-sectional data can identify a potential relationship between the environment and health at one point in time, the length of exposure to environment is unknown, preventing researchers from assessing causality between environmental factors and health.

The aim of this paper is to investigate longitudinal relationships between proximity to and density of retail food outlets around schools and adolescent reported diet in the 2001 and 2005 waves of the Research with East London Adolescents: Community Health Survey (RELACHS). We analysed these data to answer two broad questions: 1) has there been a change in the prevalence of retail food outlets around schools at each wave; and 2) are changes in adolescent diet longitudinally related to changes in the local food environment.

## Methods

The RELACHS study is a school-based longitudinal survey over three time points, administered to adolescents in 2001, 2003 and 2005. Ethical approval for the study was obtained from the East London and City Local Research Ethics Committee. All children provided informed written consent. Parents and guardians had an opt-out process whereby they only returned a signed form if they did not want their child to take part, providing informed consent. The area included in the study covers three London boroughs: Newham, Hackney and Tower Hamlets and is characterised by a high level of material and social deprivation relative to the rest of England. This paper focuses on data collected in 2001 and 2005 (diet variables were not included in the 2003 survey). The target student population were 11–12 years old (Year 7) at baseline in 2001.

Table 1 provides an overview of the sample student population at the beginning of RELACHS and in 2005. A total of 30 schools participated at baseline [32,33] randomly selected from the London boroughs of Tower Hamlets, Hackney and Newham. Twenty-nine schools had a food outlet located within 800 metres of the school and were eligible for this study, providing a sample of 1382 participants in Year 7 (Table 1). Data collected in 2005 followed up students who were in Year 7 at baseline only as the older cohort had left school. These participants were 15–16 years of age in 2005 and included 1023 respondents, of which 757 were eligible for longitudinal analysis, having also completed the survey in 2001. From the sample of 757, many of the students were missing one or more diet responses necessary for the planned analysis, and 11 did not include FSM status. No one diet variable (i.e., fruit and veg, breakfast, fizzy drink consumption) at either time point was missing from more than 15% of the sample, however, the overall pattern of missing data led to a substantial reduction in sample size. Listwise deletion of those respondents who were missing data on either FSM or diet reduced the complete case sample size to 524 (Table 1).

**Table 1:** Descriptive characteristics of RELACHS participants

**2001 younger group**	**(n=1382)**	**N**	**%**
Free School Meal eligible	Yes	604	43.7
	No	597	43.2
	missing	181	13.1
Gender	Male	691	50
	Female	691	50
		mean	sd
Healthy Diet Score (n=1325)		6.6	2.8
Unhealthy Diet Score (n=1295)		13.7	3.7
		mean	sd
Age (years, n=1227)		12.2	0.3
Imputed for 2005	(n=757)	N	%
Free School Meal eligible	Yes	376	49.7
	No	381	50.3
Gender	Male	357	47.2
	Female	400	52.8
		mean	sd
Healthy Diet Score		5.5	4.1
Unhealthy Diet Score		13.2	2.8
		mean	sd
Age (years)		16.1	0.3
Complete Case for 2005	(n=524)	N	%
Free School Meal eligible	Yes	235	44.8
	No	289	55.2
Gender	Male	222	42.4
	Female	302	57.6
		mean	sd
Healthy Diet Score		5.4	2.7
Unhealthy Diet Score		12.9	4.2
		mean	sd
Age (years)		16.1	0.3

To maximise our statistical power, we used multiple imputation in SPSS 19.0 to impute missing FSM data (Table 1) and the individual diet variables described below. We ran 15 imputations using all variables included in the later statistical analysis as predictors for missing diet data: age, gender, eligibility for free school meals (FSM) [34]. Eligibility for FSM provided a proxy indicator for individual socioeconomic status because the criteria for FSM includes family income and has been shown to accurately represent adolescent socioeconomic status in the UK [35,36]. Table 1 summarises the distribution of missing demographic data, attrition and the resulting imputed dataset.

Healthy diet:

‘Before going to school how often do you have breakfast at home or school breakfast club?’

Scores are assigned as follows:

Every day = 3

3-4 days/week = 2

1-2 days/week = 1

Never or hardly ever = 0

And a derived portion of fruit and vegetable portions eaten each day based on the follow questions:

‘About how many lots of fruit do you eat in a day?’

[‘How many lots’ means ‘how many portions’ (e.g. one apple/small bunch of grapes)]

(None, 1, 2, 3, 4, 5 or more)

‘About how many lots of vegetables do you eat in a day?’

The score is calculated from the number of portions of fruit and vegetables reported to be consumed in a day added to the breakfast score.

Unhealthy diet:

‘How often do you eat or drink the following?’

Crisps or savoury snacks

Sweets, ghee sweets or chocolate

Biscuits

Fried food, chips, samosas or bhajis, or Fried English Breakfast

Fizzy Drinks (e.g. Coke)

(More than once a day, once a day, at least once a week, rarely, never)

Scored as follows for each category, then summed for an overall unhealthy diet score:

More than once a day = 4

Once a day = 3

At least once a week = 2

Rarely = 1

Never = 0

The RELACHS questionnaire includes questions on dietary habits in 2001 and 2005. A summary of the questions used to classify respondent diets are shown above. The diet outcomes were scored as ‘healthy’ and ‘unhealthy’, following earlier work on this dataset [37]. Two aggregate diet variables were created by classifying each set of responses to all of the relevant questions for each measure (n=3 for healthy, n=5 for the unhealthy diet variable). To quantify a healthy diet, participants were assigned higher values for eating breakfast more frequently and eating more portions of fruit and vegetables. Scores for fruit and vegetable consumption correspond to the total portions of fruit and vegetables consumed in a day. For example, if a student never ate breakfast (scoring zero) and consumed two portions of fruit and one portion of vegetables a day, the healthy diet score would be three (0 + 2 + 1 = 3). The unhealthy diet score is calculated from the responses to five questions with a value assigned to each response on the Likert scale. The unhealthy diet score is the sum of the values assigned to the five answers provided about consumption frequency of selected food items. The healthy diet variable included a range of 0 (least healthy) to 13 (most healthy) based on the maximum value in the sample. The unhealthy diet variable was treated similarly and included a range of values from 0 to 20.

### Food environments

Data on the local food environment were collated from telephone directory listings archived at the Tower Hamlets Local History Library and Archives. The local councils were contacted with requests for environmental health registers which include all locations where food is sold. Unfortunately when the registers are updated previous versions are overwritten so records from 2001 and 2005 were unavailable. Data were not available electronically either online or from a commercial data provider (yell.com). For 2001 the Thompsons phone directory, combined with the Yellow Pages covered all postcode sectors included in the study area. In 2005, retail food data for the entire study area were available from the Yellow Pages directory only. Phone directories are not a perfect source of food environment data [38,39], however, previous research has employed similar approaches to create historical food environment datasets [29,30].

Entries from all phone directories were recorded, compiled and de-duplicated. Electronic web searches (Google) using the business name, street address and postcode sector (i.e., E1) listed in the phone directories allowed us to identify the complete unit postcodes (i.e., E1 4NS). The results were recorded in an Excel spreadsheet which listed the business name, address and full postcode.

Postcodes are the most precise and pragmatic measure of location available in the UK, typically including only 15 street address locations [40]. The geographic x y coordinates of each postcode were attached to all food outlets using look-up tables available online from UK Borders [41]. This allowed the food outlet locations to be projected in the GIS. All food outlet data were again checked for duplicate records. The accuracy of postcodes of 10% of the sample was verified by a second researcher, using internet searches with the business name, street address and postcode sector as described above to ensure the same results were found, which they were. Schools were entered into the GIS using the same method of attaching x y coordinates to each postcode.

Food outlets were classified by the section of the phone directory where they were listed (takeaway, grocer, supermarket). The food outlet type was grouped into either takeaway (fast food) or grocer/supermarket/convenience store. This aggregation was necessary to be consistent with phone directory headings. In addition, we were unable to differentiate all traditional supermarkets or grocery stores from smaller corner shops and convenience stores on the basis of shop name.

Network analysis in a GIS was used to identify how many shops of each type were located within 400 m and 800 m road network distance from the schools. These measures represent the distance easily walked in five or ten minutes and are frequently applied as regions of influence for predominantly pedestrian populations in food access research, including around schools [11,13-16]. The study of student purchasing patterns identified that shops on the main transport routes to schools were most often used, further justifying the distance measures for this urban student population [21]. Minimum and median distance to each outlet type within the two distance buffers in 2001 and 2005 was calculated. The minimum distance provides an indication of the closest food outlet within the shortest distance to the school, which will be the same for both distance buffers if the value is below 400 metres. The median distance provides an measure of the range of distances students must travel within the 400 or 800 metre road network buffers, and preferred to measures of mean distances for non-normally distributed data [4]. Occasionally a school shared a postcode with a food outlet so the distance is reported as 0 metres.

### Analysis

Statistically significant changes to the mean healthy and unhealthy diet scores over time were assessed for the imputed data using Wilcoxon signed-rank test. This is a nonparametric test for paired data. The same statistical test was applied to the data on food environments around schools in 2001 and 2005: counts of total outlets, median distance to grocer or takeaway and minimum distance to a grocer or takeaway.

Bivariate relationships between measures of food access and imputed sociodemographic characteristics (age, gender, FSM eligibility) with diet scores were assessed. Diet outcomes were analysed as continuous variables and the count, median or minimum distance to a food outlet of a chosen type within the two network buffers was included for each participant based on their school location. For statistically significant relationships (p < 0.05), a generalised linear model (GLM) was then used to adjust for the demographic variables: age, gender and FSM eligibility, with the analysis clustered by school. The imputed diet data was included in the GLM using a pooled analysis in SPSS 19.0.

In addition to age, gender and FSM eligibility, earlier bivariate (Spearman’s rank correlation) analysis on the imputed dataset not reported here tested for any difference in diet, proximity or count of food outlets by school-level deprivation (see [13]) and individual ethnicity. There were no significant relationships or differences by school deprivation, which is likely due to the similar levels of deprivation in each school population. Student ethnicity was not significantly related to diet outcomes in this sample and excluded from further analysis.

In the GLM analysis the outcome variable was set as the diet outcome (healthy or unhealthy diet score) in 2005. Models included the corresponding (healthy or unhealthy) diet outcome in 2001 as a continuous predictor variable to control for baseline diet score within individuals. All models were adjusted for demographic variables from 2005 data. Each model then included either the distance to food outlet measures for 2005 (minimum or median) or the count of outlets by type to explore the effect of access on adolescent diet scores.

## Results

The initial results of the GIS analysis reflect some change in the food environments near schools between 2001 and 2005. The number of takeaways within 400 metres of a school was similar in both years, but by 2005 there were 115 more convenience stores/grocers within 800 metres of a school (Table 2). There is an increase in the median distance to convenience stores/grocers, though the minimum distance to grocers within both buffer areas decreased by an average of 15.9 metres. Comparing all of the food environment data around schools from 2001 to 2005, the only statistically significant change in food environment is the count of grocery/convenience stores within 800 metres of a school (p<0.001). All other pairs of data (median or minimum distance to an outlet at either 400 or 800 metre network buffers) were not significantly different between 2001 and 2005.

**Table 2:** Retail environment change: count and distance from nearest schools, 2001 to 2005

	**400 m**				**800 m**			
	**Distance in metres**				**Distance in metres**			
	**count**	**median**	**minimum**	**range**	**count**	**median**	**minimum**	**range**
**Grocer**								
2001	51	283.8	54.2	340.2	151	516	54.2	741.2
2005	68	290.3	38.3	356.2	266	580.5	38.3	760.1
Change, 2005 to 2001	17	6.5	−15.9	16	115	64.5	−15.9	18.9
**Takeaway**								
2001	43	272.2	0	396.5	180	529.3	0	791.6
2005	45	282.1	0	393.9	170	507.7	0	795.4
Change, 2005 to 2001	2	9.9	0	−2.6	−10	−21.6	0	3.8

The change in diet score between 2001 and 2005 for the 757 respondents with longitudinal data is reported in Table 3. Nearly all mean diet values decreased over the four years, with the exception of fried food. The mean scores decreased for both healthy (mean decrease of 1.10) and unhealthy (mean decrease of 0.48) diets. The change in both healthy and unhealthy diet scores over time is statistically significant (p< 0.001). This decrease in mean scores for both types of diet is explained by the different variables which comprise each diet ‘type’. The mean changes to both breakfast and fruit/vegetable consumption cause the healthy diet score to decrease. Similar negative mean changes in four of the five individual variables in the unhealthy diet score ensures that the overall score shows a mean decrease.

**Table 3:** Longitudinal changes to mean diet scores (2005 – 2001)

	**Healthy diet**	**Unhealthy diet**	**Breakfast**	**Fizzy drink**	**F&V**	**Crisp**	**Sweet**	**Biscuit**	**Fried food**
Mean	−1.10	−0.48	−0.54	−0.21	−0.55	−0.17	−0.08	−0.08	0.06
SE	0.12	0.16	0.05	0.05	0.11	0.05	0.05	0.05	0.05

Table 4 shows the results of adjusted GLM analyses on the diet outcome at 2005. There were no statistically significant relationships between the count of food outlets and diet scores. Healthy diet scores are positively correlated with the minimum distance to grocery stores, within both 400 and 800 metre buffers. Unhealthy diet scores are negatively correlated with the median distance to takeaways within 400 m, and the minimum distances to grocers within 800 m and takeaways at both distances. The strongest relationship is observed between the healthy diet scores for 2005 and the positive correlation with median distance to grocers within 800 metres (0.003, 95% CI 0.001 to 0.005). All relationships between outlet access and healthy diet were positive, suggesting that adolescents who are likely to travel further from school to reach a food outlet reported a healthier diet in terms of fruit and vegetable and breakfast consumption. The small significant relationships between proximity to takeaways and unhealthy diets indicate that students who must go further from school to reach a takeaway have a less unhealthy diet.

**Table 4:** Adjusted estimates for the effect of distance to food shops on diet

**Median distances from schools to outlet types**				
Diet type	Grocer, 400 m	Grocer, 800 m	Takeaway, 400 m	Takeaway, 800 m
Healthy diet		0.003 (0.001, 0.005)	0.002 (0.001, 0.004)	
Unhealthy diet			−0.003 (−0.006, -0.001)	
**Minimum distances from schools to outlet types**				
Diet type	Grocer, 400 m	Grocer, 800 m	Takeaway, 400 m	Takeaway, 800 m
Healthy diet	0.003 (0.001, 0.006)	0.002 (0.000, 0.003)	0.002 (0.001, 0.004)	
Unhealthy diet		−0.001 (−0.003, 0.000)	−0.003 (−0.006, 0.000)	−0.002 (−0.004, 0.000)

## Discussion

The aim of this paper was twofold: to assess how the retail food environment around schools may have changed over four years in a relatively deprived area of the UK and to determine whether the food environment near secondary schools has any influence on adolescent diets. This research reflects the expressed need for greater clarity of the role local environments may have in shaping individuals’ health as reported in the Marmot Review [42]. The importance of local government’s scope to respond to identified inequalities is explicit in a follow-up to the Marmot Review and provides context for these results [43].

Retail environment adjusted over the study period. The count of takeaways within 400 metres of schools was similar over time, while there was a small increase in the number of grocers within the same distance. The difference in food environment was greater within the wider range of 800 metres: a decrease in the number of takeaways and a marked increase in convenience stores or grocers from 2001 to 2005, the only statistically significant observed change to the food environment. This study is unusual in evaluation of retail food environments over time, with only a few previous studies including retail change [29-31]. However, the necessity of using phone directories from two different companies (Thompson’s and Yellow Pages) may partly explain the change in retail food environment. It may be that more businesses chose to advertise in the 2005 telephone directory due to cost or similar reasons.

Results from our analysis indicate that changes in the local food environment may have some marginal effect on the diets of adolescents in this study. Healthy diet scores showed positive parameters for minimum distance to stores within both buffers, and median distance within 800 m. These associations may offer some evidence that a slightly greater distance between school and the nearest grocer/convenience store may contribute to more frequent breakfast, fruit and vegetable consumption. We have combined outlet types (grocers, convenience stores and supermarkets) which are often distinct in other studies [18], however, the type of food students are likely to purchase from any retail outlets on the school commute is more likely to be included in the ‘unhealthy’ diet score such as crisps, fizzy drinks and chocolate [21]. This offers some explanation for the positive relationship between distance to grocers and healthy diet scores.

Distance to takeaways had a marginal negative association with unhealthy diet; if students had to walk further to the takeaway they may have a less unhealthy diet. This is similar to results from longitudinal research in the US, where access to fast food in the residential neighbourhood was not related to consumption. The authors of the US study suggest that young adults may be more likely to purchase fast food nearer to work or school, which may partially explain their null results [28]. One further study concluded that among low-income men proximity to fast food near home increased consumption, though access to grocery stores had no discernible effect on diet [25]. We found no evidence that distance to fast food increased the unhealthy diet score, but there was no question in RELACHS that explicitly queried fast food consumption, the most similar variable was fried food.

The statistical model results in our research differ slightly from previous cross-sectional research which found no significant association between diet and proximity to retail outlets in the UK [44] and New Zealand [4]. Most studies which report any statistically significant relationship between physical access and resident diet are based in materially deprived areas or populations of the US [9,45,46]; the geographic area in this research is also highly deprived. It may be that proximity is more important in deprived populations or areas, where physical access is limited by walking or public transport. As our study population is adolescents the effect of proximity may be greater since they cannot drive.

Few studies have reported the influence of food outlets near schools and peer groups on adolescent diet [16,21], and limited opportunities are available to assess diet in relation to food environment over time. Often there is no clear relationship between the food environment and student diet as measured by surveys/FFQs, which may result from cross-sectional study design, small numbers of participants, or uncertainty in reporting of diet [11].

The results reported in this paper are relatively unique in a UK context and may be partially explained by the study design. The longitudinal data, which allowed us to explore the changing retail environment as well, could explain the small significant association between diet score and proximity to food outlets within both distance buffers. In addition, the point of origin was shifted from place of residence to the location of participants’ schools. This represents an alternative food environment to those measured in most previous research, building on recent work which incorporates food environments near to or within schools [11-14,16,47]. A US study surveyed 349 adolescents to gather information about discrete elements of their diets and considered how diet might be influenced by the local food environment around their schools. They concluded that some aspects of diet were influenced by the local food environment, such as sugar sweetened beverages, while fruit and vegetables consumption was not [48]. Given the small effects reported in our study and small or null results in similar research, residual confounding may explain our marginal findings.

We acknowledge that in previous research there has been a division of supermarket/grocery stores and corner/convenience shops [11,47]; this was not feasible for this research. The types of foods purchased by students during the school – home journey (crisps, chocolate) [48] are available at all store types, with evidence that students shop for better prices which may be found at supermarkets [21]. Though grocers are often used as a proxy for ‘healthy’ food options, they also stock the items included in the unhealthy diet score: fizzy drinks, crisps, sweets and biscuits. If the adolescents were relying on these stores to purchase unhealthy food items as they travel to or from school, the slightly greater distance may have contributed to the decrease in ‘unhealthy’ food items consumed in the second wave of the longitudinal sample, though there were many more grocers/convenience stores in 2005. We cannot assume that takeaways are the only source of ‘unhealthy’ food, or that consumers will avoid the fizzy drinks and crisps at the grocers (see also [49]). Further qualitative research on individual shopping behaviour and purchasing decisions will give us insight into food outlet choice and the variety of items selected in different store formats [50], which will enhance later research.

Limitations to this study include the inability to account for what adolescents may purchase and consume within the schools or bring from home; the food environment information is restricted to outlets outside of the school. The data on food outlets was limited to information available from phone directories because the local council-held food registers are updated regularly, without keeping a record of previous businesses which have closed. One challenge with phone directories as a data source is the lack of specific code to indicate the type of food outlet other than generic self-identification of grocer or supermarket. The historical food environments only include those businesses that are listed in the phone directories. However this is a similar limitation to most food access research [51,52]. A recent validation of several data sources for food outlet locations in Newcastle, UK concluded that although the Yellow Pages reported more food outlets that were not actually present on the ground, most of these were either pubs/bars or restaurants [38]. Neither of these categories is used in this paper. We were required to use two different phone directories to provide total geographic coverage in 2001, the Yellow Pages did not include all of the areas in our study. The mismatch in datasets may introduce some error from the slightly different data sources. The Thompsons’ directory was unavailable in 2005 however the 2005 Yellow Pages included the entire study area at the second time point.

Further challenges with interpretation of the results can be described as uncertainty. While we can refer back to research describing adolescent purchasing behaviour around schools, data was not collected for this study. We do not have food purchasing data about the home food environment, which may have a substantial impact on overall diet for the participants, especially if they are from lower income homes with limited money for out-of-home food purchases. One part of the healthy diet is consumption of breakfast, though the nature of each respondent’s breakfast is unclear. If breakfast is a high-fat sausage roll which is not included in any of the unhealthy diet variables, then the scores may not reflect diet as accurately as we intend.

Finally, there was attrition in the respondent population between 2001 and 2005 as described in Table 1. We addressed this loss of sample size by imputing the missing diet variables which are included in the diet scores as outlined in the methods section. However, we acknowledge that the imputation may introduce some bias in the final results.

## Conclusion

There are very few published studies which analyse longitudinal data on both diet outcomes and local food environments, and there is an absence of significant results from cross-sectional analysis of food environment and diet [11,16]. The research presented in this paper adds to the literature on longitudinal studies of diet and environment, the only type of studies which may draw a direct link between environment and health, due to exposure over time. The Marmot Review [42] reported on the need for a greater understanding of the influence social inequalities have on health, and a more recent policy report states the need for greater intervention at the local level to improve public health [43]. Here we present results which suggest that in East London there is a weak association between the proximity of retail food outlets to secondary schools and adolescents’ diets. The results presented in this paper warrant further exploration over a larger and more diverse geographic area, to identify if this potential relationship between food environment and diet is present in other areas.

Proximity is not the sole factor in food purchasing behaviour, and a challenge with analysing local food environments is the implicit assumption that individuals frequent food outlets near home; future work needs to measure multiple and alternative food environments. In addition, there should be more careful consideration given to the types of foods available in food outlets. The marginal significant relationships between proximity and diet which we identified should be treated with caution, however, results suggest that adolescents have healthier diets when their schools are located further from retail food outlets regardless of type. We must move beyond the simplistic approach of healthy food availability represented by grocers and consider the full range of food items stocked in these outlets.

## Abbreviations

BMI: Body Mass Index; FSM: Free School Meal; GIS: Geographic Information Systems; GLM: Generalised Linear Model; RELACHS: Research with East London Adolescents: Community Health Survey.

## Competing interests

The authors declare that they have no competing interests.

## Authors’ contributions

DS wrote the initial draft, carried out the mapping, data analysis and the literature review. SC contributed to the conception of the paper and discussion of the analysis. CC and SS assisted with data acquisition and contributed to the conception of the paper. All authors contributed to successive drafts of the paper, read and approved the final draft. DS is the guarantor. All authors read and approved the final manuscript.

## Pre-publication history

The pre-publication history for this paper can be accessed here:

http://www.biomedcentral.com/1471-2458/13/70/prepub
